# David Walter Millard, FRCPsych, MD, MA (Mus)

**DOI:** 10.1192/bjb.2021.85

**Published:** 2022-04

**Authors:** Rupert McShane

Formerly Consultant Psychiatrist, Oxfordshire Mental Healthcare Trust, and Lecturer in Applied Social Studies, University of Oxford, UK

**Figure:**
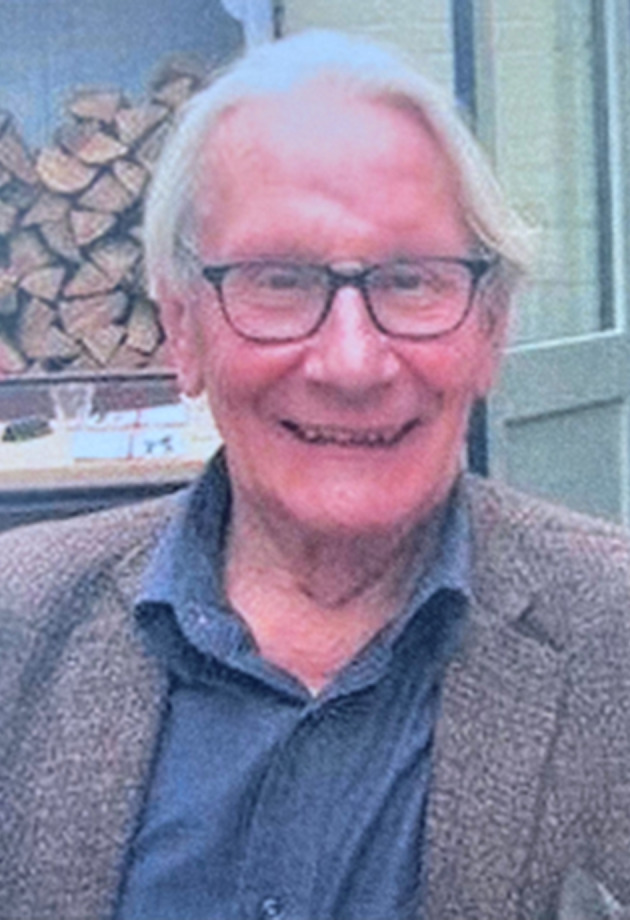

David Millard, who died on 13 January 2021 aged 89, had a varied career as a practitioner, teacher, academic and historian. The idea that a patient's social context could be altered in such a way that it enhances recovery from mental illness was at the heart of his work. The practical manifestation of this idea was the therapeutic community movement. David's interest in what makes groups and institutions tick resulted in a career that was framed by psychiatry, but which, in the middle period, took what was, at the time, an unconventional multidisciplinary path.

David was born on 9 February 1931 in Gloucestershire to Walter, a biology teacher, and Gladys Jarvis, a nurse (and a descendent of Thomas Hardy). One of David's school reports berated him for spending so much time on music, but his parents’ occupations were influential in his choice of medicine. Following qualification from the University of Birmingham Medical School, he had intended to pursue a career in surgery, but Harold Macmillan and Suez intervened and a short service commission as a doctor in the Royal Army Medical Corps followed. On return, his career changed direction and he embarked on psychiatric training at Rubery Hill Hospital in Birmingham. There he experienced the therapeutic community run by John Yerburgh, which shaped his future interests and career choices. He moved to Sir William Trethowan's professorial unit at the Queen Elizabeth Hospital in Birmingham, where he established a therapeutic community on Ward North 5A. Returning to Rubery as a young consultant, he was instrumental with others in building a third therapeutic community. However, after just 4 years, in 1970, his enthusiasm for the workings of multidisciplinary teams, growing academic interests and enjoyment of teaching led him to take the unusual step of relinquishing his consultant post in Birmingham to become Lecturer in the Department of Applied Social Studies at the University of Oxford. This reflected his interest in bringing psychiatry and social work together. The students were trainee social workers and probation officers. A devout Christian, he also taught Psychology of Religion for the Faculty of Theology and was for 20 years a Council Member and chair of the Institute of Religion and Medicine.

Through his continuing interest in the new and evolving Association of Therapeutic Communities (founded in 1972), David became the inaugural convener of its research group, followed by 8 years as joint editor of the *International Journal of Therapeutic Communities*. A prolific contributor to this and related fields, his MD thesis *Collected Writings on the Therapeutic Community* (1995) included an important contribution on the life and work of Maxwell Jones. This was reprinted in *150 Years of British Psychiatry 1841–1991: The Aftermath* (1996), edited by Hugh Freeman and German Berrios.

In 1979, under Sir Richard Doll's wardenship, David was invited to be one of the 18 Founding Fellows of Green College Oxford. He held posts as Senior Tutor and later Dean of Degrees. His devotion to the College was to last for the rest of his life. On retirement he was made an Emeritus Fellow of the College.

During his years teaching in the Department of Applied Social Studies, David maintained his clinical interests as an honorary consultant in Michael Gelder's newly formed professorial unit at the Warneford Hospital in Oxford, where he provided a psychodynamically informed out-patient service, including teaching medical students in the clinic. Gelder's style of rigorous structured exploration of psychiatric diagnosis and the ambitiousness of academic medicine was not really to David's taste – but he enjoyed seeing patients too much to give up clinical work.

He retired from academic work in 1991 but continued his connection with NHS psychiatry, taking on a substantive post as consultant in old age psychiatry in 1994, which was to last for 6 years.

In Oxford his wide interest in social processes within institutional contexts led to his involvement in the governance of local organisations concerned with the elderly and with delivering often innovative services to psychologically vulnerable people in the community. Always interested in the most disadvantaged, David nurtured organisations that looked for restorative pathways, such as those for the homeless and people involved with the criminal justice system.

David was a careful, thoughtful listener. His wise advice – ‘If it's not clear, get more information’ – lies at the antithesis of decisive medical action in the absence of certainty, but was a constant reminder to go the extra mile for the patient. He led, but did not drive his teams to make better, broader, decisions. His espousal of flat hierarchy, drawn from the same humility that drew him to therapeutic communities, endeared him to staff of all disciplines.

An able cellist, pianist and musician with a keen interest in historical instruments, including constructing his own clavichord, David obtained an MA in Music following retirement. His dissertation, on musical feminine endings – in which an additional unexpected step is added and the penultimate chord is the strongest – betrayed his deep interest in the psychology of resolution.

He married Heather, by whom he had two children (Julian and Hilary). They divorced in 1976. He then married Sheila, a psychiatric social worker and group analyst, whose attention he attracted by his performance in role-play as an elderly grandmother at a conference.

In his later years he suffered from vascular dementia. It was fitting that, in his final illness, his wife was supported in his care by doctors, nurses and institutions whose development David had influenced: he reaped as he had sowed.

He is survived by Sheila, his two children, his two step-children (Peter and Laura) and his sister Ann.

